# Robustly Engineering Thermal Conductivity of Bilayer Graphene by Interlayer Bonding

**DOI:** 10.1038/srep22011

**Published:** 2016-02-25

**Authors:** Xiaoliang Zhang, Yufei Gao, Yuli Chen, Ming Hu

**Affiliations:** 1 Institute of Mineral Engineering, Division of Materials Science and Engineering, Faculty of Georesources and Materials Engineering, RWTH Aachen University, 52064 Aachen, Germany; 2 School of Architecture & Civil Engineering, Shenyang University of Technology, Shenyang 110870, China; 3 Institute of Solid Mechanics, Beihang University (BUAA), Beijing 100191, China; 4 Aachen Institute for Advanced Study in Computational Engineering Science (AICES), RWTH Aachen University, 52062 Aachen, Germany

## Abstract

Graphene and its bilayer structure are the two-dimensional crystalline form of carbon, whose extraordinary electron mobility and other unique features hold great promise for nanoscale electronics and photonics. Their realistic applications in emerging nanoelectronics usually call for thermal transport manipulation in a controllable and precise manner. In this paper we systematically studied the effect of interlayer covalent bonding, in particular different interlay bonding arrangement, on the thermal conductivity of bilayer graphene using equilibrium molecular dynamics simulations. It is revealed that, the thermal conductivity of randomly bonded bilayer graphene decreases monotonically with the increase of interlayer bonding density, however, for the regularly bonded bilayer graphene structure the thermal conductivity possesses unexpectedly non-monotonic dependence on the interlayer bonding density. The results suggest that the thermal conductivity of bilayer graphene depends not only on the interlayer bonding density, but also on the detailed topological configuration of the interlayer bonding. The underlying mechanism for this abnormal phenomenon is identified by means of phonon spectral energy density, participation ratio and mode weight factor analysis. The large tunability of thermal conductivity of bilayer graphene through rational interlayer bonding arrangement paves the way to achieve other desired properties for potential nanoelectronics applications involving graphene layers.

Graphene, the first two-dimensional atomic crystal available to us, has attracted great attentions due to its supreme mechanical^1^, electronic^2^ and optical^3^ properties. Except for these properties, thermal^4^,^5^ and thermoelectric^6^ properties of graphene are also very fascinating. Experiments have shown that the thermal conductivity of graphene is as high as about 5000 W/mK 5, which makes graphene very promising for thermal management applications such as heat dissipation in electronics. In electronics, especially for nanoelectronics, interfacial thermal resistance is a key factor that affects the heat dissipation in devices and researchers have shown that the interfacial thermal transport can be largely enhanced by using graphene-based nanocomposites as thermal interface materials^7^. In addition to the heat dissipation applications, due to its extremely high electrical conductivity, graphene has also been explored to be used as thermoelectric materials by largely reducing its thermal conductivity by varieties of functionalization, such as constructing graphene nanoribbons^6^, hydrogenation^8^, defects^9^, doping^10^, and so on. As demonstrated above, the extremely high intrinsic thermal conductivity of graphene can be used for heat dissipation and extremely low thermal conductivity can be used for thermoelectric energy conversion. Therefore, tuning its thermal conductivity to be different range is of great importance for varieties of applications.

In addition to monolayer graphene, there has been a growing interest in bilayer and multilayer graphene, which have also shown promising electronic^11^ and thermal^7^ properties. For bilayer and multilayer graphene, generally the interlayer interactions are considered to be weak van der Waals forces, thus, as compared with covalent bonding (Fig. 1) the interlayer phonon coupling with van der Waals forces is relatively weak and has a relatively small effect on the in-plane thermal transport properties of graphene structures. Interestingly, a scanning tunneling microscopy study and *ab initio* total energy calculations have identified that the interlayer sp^3^ bonds in graphite can be formed by femtosecond-laser excitation^12^, which will generate strong interlayer phonon coupling and have a significant effect on the in-plane thermal conductivity of the graphene structures. Non-equilibrium molecular dynamics simulations^13^ have shown that the randomly distributed interlayer sp^3^ bonds can lead to monotonic reduction of the thermal conductivity of bilayer graphene as a function of interlayer sp^3^ bond concentration. However, in fact, interlayer bonding (Fig. 1) has a complicated effect on the in-plane thermal conductivity of bilayer two-dimensional structures, depending on not only the interlayer bonding density but also the detailed topological configuration of the interlayer bonds^14^.

In this paper, by performing equilibrium molecular dynamics (EMD) simulations, we investigated the effect of interlayer sp^3^ bonding on the thermal conductivity of bilayer graphene, especially we compared the randomly and regularly bonded bilayer graphene structures. We demonstrate that the thermal conductivity of randomly bonded bilayer graphene decreases monotonically with the increase of interlayer bonding density. However, non-monotonic interlayer bonding dependence of thermal conductivity of regularly bonded bilayer graphene is observed. The underlying mechanism for this counter-intuitive phenomenon is explored in detail by the phonon spectral energy density, participation ratio and mode weight factor analysis.

## Simulation Results

To get a converged size for calculating the thermal conductivity of the interlayer sp^3^ bonded bilayer graphene structures, we first investigated the size effect on the thermal conductivity of regularly bonded bilayer graphene with interlayer sp^3^ bonding density of 6.25%, as shown in Fig. 2. We can see that the thermal conductivities of bilayer graphene in both armchair and zigzag directions are converged when the supercell size reaches 4 unit cells. This converged supercell size is used for the thermal conductivity calculations of all the sp^3^ bonded bilayer graphene structures we studied. Note that the thermal conductivities are different for armchair and zigzag directions, which should be due to the difference of the detailed topological arrangement of the interfacial bonding in armchair and zigzag directions. The thermal conductivity of the regularly bonded bilayer graphene with interlayer sp^3^ bonding density of 6.25% converges for simulation box size of ~5 nm and 10 nm for armchair and zigzag direction, respectively. This is comparable with the convergence size of 10 nm for single layer graphene from our separate simulation run. Note that the results shown in Fig. 2 are using the Green-Kubo method (EMD), where the size effect is generally small. In contrast, the size effect in the non-equilibrium molecular dynamics (NEMD) simulations for normal nanostructures is quite strong.

The thermal conductivity of bilayer graphene as a function of interlayer bonding density is presented in Fig. 3. For comparison we also studied the cases of random and regular bonding and the results are also shown in Fig. 3. It is unexpectedly seen that with the increase of interlayer sp^3^ bonding, the thermal conductivity of randomly bonded bilayer graphene decreases monotonically. However, the thermal conductivity of regularly bonded bilayer graphene changes non-monotonically. Especially, there are two obvious peaks at the interlayer bonding densities of 6.25% and 12.5%. The results suggest that the thermal conductivity of sp^3^ bonded bilayer graphene depends on not only the bonding density, but also the detailed topological arrangement of the interfacial bonding. The thermal conductivity of sp^3^ bonded bilayer graphene falls in a broad range between several tens and a few hundred W/mK, indicating that the interlayer bonding is an effective way to manipulate the thermal transport properties of bilayer graphene, which could be very beneficial for varieties of potential applications.

## Discussion

Researchers have shown that covalent bonds will influence the thermal conductivity disregard whether there is a second layer^15^, which helps to explain the lower thermal conductivity of bilayer graphene after covalent bonds are added. However, it is counter-intuitive that the bonds with a periodic pattern can have thermal conductivity much larger than those distributed randomly and the non-monotonic thermal conductivity changing trend of the regularly bonded bilayer graphene is also worth to explore.

In order to explore the mechanisms for the non-monotonic thermal conductivity results of the regularly bonded bilayer graphene and the generally higher thermal conductivity as compared with the randomly bonded structure, we investigated the phonon mode dependent thermal conductivity of bilayer graphene by performing spectral energy density (SED) analysis. First, the phonon normal modes are obtained by^16^


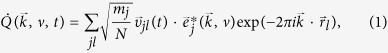


where the eigenvector 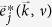 was obtained by harmonic lattice dynamics calculation using the PHONOPY software^17^. Then the spectral energy density is calculated by^18^


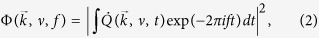


and the phonon lifetime is obtained by fitting the Lorentzian function^19^. Finally, the phonon mode dependent thermal conductivity of the bilayer graphene can be derived from the phonon Boltzmann transport equation (BTE) under the relaxation time approximation^20^,^21^


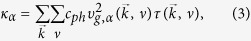


where 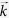 is the wave vector in the first Brillouin zone, *ν* is the phonon branch, *κ*_*α*_ denotes the thermal conductivity in *α* direction, *c*_*ph*_ is phonon volumetric specific heat calculated by 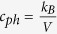, *k*_*B*_ is the Boltzmann constant, *V* is the system volume, *υ*_*g,α*_ is *α*-component of the phonon group velocity, *τ* is the phonon lifetime. Note that the above phonon SED method has been successfully used by us to perform the phonon mode analysis for a wide variety of nanostructures, e.g., two-dimensional silicene^22^, boron nitride (BN)^14^, and one-dimensional graphyne nanotubes^23^. Other researchers have also investigated the phonon lifetime of graphene using the SED method^24^ or an exact numerical solution of the Boltzmann transport equation^25^.

The frequency dependent phonon lifetimes of bilayer graphene (interlayer bonding density of 6.25%) with randomly and regularly bonded structures are compared in Fig. 4(a). We can see that the low-frequency (<5 THz) phonon lifetimes for randomly and regularly bonded bilayer graphene are nearly the same, indicating that the low-frequency phonons are not sensitive to the detailed interfacial bonding configuration. This is understandable considering that the low-frequency phonons usually have a far longer wavelength than the characteristic distance between the bonded sites and can travel easily through the structures without scattering. However, for medium-frequency (5–30 THz) and high-frequency (30–50 THz) phonons the lifetimes are greatly larger for the case of regular bonding than that of randomly bonded structure, especially for the medium-frequency phonon modes, which have a dominant contribution to the higher thermal conductivity of the regularly bonded bilayer graphene than the randomly bonded structure, as evidenced by Fig. 4(c). Furthermore, the square of phonon group velocity in Eq. (3) as a function of frequency is shown in Fig. 4(b). It is clearly seen that, throughout the entire frequency range, the velocity square for the case of regularly bonded bilayer graphene is much higher than that for randomly bonded structure. To evaluate the relative importance of the contributions of phonon lifetime and velocity square to the enhanced thermal conductivity of the regularly bonded bilayer graphene as compared to the randomly bonded structure, we exchanged the square of the group velocity and the phonon lifetime between the randomly and regularly bonded bilayer graphene structures. That is, we define the following four types of thermal conductivity:


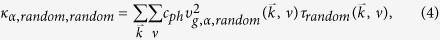



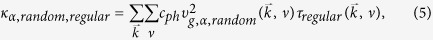



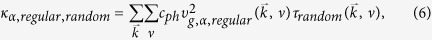



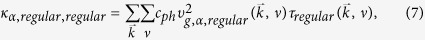


Using the above definition for thermal conductivity, we obtained four results of the thermal conductivity of bilayer graphene with interlayer bonding density of 6.25%: 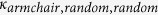 = 21.77 W/mK, 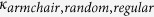 = 27.19 W/mK, 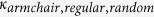 = 93.90 W/mK, 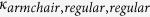 = 145.81 W/mK. Note that here we just used the harmonic group velocity and did not consider the effect of the shifted anharmonic frequency on the group velocity which has been naturally included in the SED calculation, so there is a little difference between the above results and the SED results. From the above results, we can see clearly that the augmentation of the square of phonon group velocity is the dominant mechanism for the enhanced thermal conductivity of the regularly bonded bilayer graphene compared to the randomly bonded bilayer graphene. The frequency dependent thermal conductivity of the sp^3^ bonded bilayer graphene (not shown for brevity) show the obviously different feature between the randomly and regularly bonded bilayer graphene. To identify the phonon frequency dependent relative contributions of the overall thermal conductivity, we also calculated the accumulated thermal conductivity percentage as a function of frequency for both the randomly and regularly bonded bilayer graphene, as shown in Fig. 4(c). For randomly bonded bilayer graphene, the low-frequency phonon modes dominate and the phonon modes with frequency lower than 5 THz contribute about 74% to the overall thermal conductivity; while for the regularly bonded bilayer graphene, the phonon modes with frequency lower than 5 THz contribute only about 32% to the overall thermal conductivity and the rest 68% of the overall thermal conductivity comes from the contributions of the higher frequency phonon modes. Therefore, compared to the randomly bonded bilayer graphene which has mainly the contributions from the low-frequency phonon modes (<5 THz, contributing 74%) and minor contributions from medium-frequency phonon modes (5–30 THz, contributing 23%), the regularly bonded bilayer graphene has contributions from both the low-frequency modes (<5 THz, contributing 32%) and medium-frequency phonons (5–30 THz, contributing 63%). For even higher frequency phonon modes, both regular and random bonding show a minor increase in the overall thermal conductivity, suggesting that the very high frequency phonon modes do not contribute much to the thermal transport in bilayer graphene. This is reasonable considering that the high frequency phonon modes are usually localized and propagate diffusively with very low phonon relaxation time [Fig. 4(a)]. Furthermore, we show the phonon mean free path (MFP) accumulated thermal conductivity in Fig. 4(d), as compared with the frequency accumulated thermal conductivity in Fig. 4(c). We can see that the maximum phonon MFP of the random topological configuration is only about 500 nm, which is far shorter than that of the regular bonded bilayer graphene structure (up to ~1400 nm). However, the long MFP phonons (from 500 to 1400 nm) in the regular bonded bilayer graphene do not contribute too much to the overall thermal transport. Instead, the short MFP phonons (less than 100 nm), corresponding to the phonon frequency range of about 5 to 30 THz (medium frequency range), contribute almost 80% of the overall heat conduction. The results are consistent with Fig. 4(c). Furthermore, as compared with the phonon MFP accumulated thermal conductivity of single-layer graphene obtained by other groups^26^,^27^,^28^,^29^, where the MFP of free standing single layer graphene ranges from 2 μm to up to 1000 μm, we can see that the presence of the interlayer sp^3^ bonds would lead to dramatic reduction in phonon MFP. Combining Fig. 4(a–d) we can conclude that the much higher thermal conductivity of the bilayer graphene with regular bonding as compared with random bonding is mainly due to the large enhancement in the group velocity of the phonon modes in the medium-frequency range (5–30 THz).

We show in Fig. 5 for the cases of bonding density of 15%, which can be compared with Fig. 4 for bonding density of 6.25%. From Fig. 4(a) with bonding density of 6.25%, we can see clearly the large difference of phonon lifetime between random and regular structures. However, from Fig. 5(a), we can see the difference of phonon lifetime is very small. Also, from Fig. 5(c) we can see that the difference of the thermal conductivity between random and regular structures with bonding density of 15% is also largely reduced, as compared to that with bonding density of 6.25%. From Fig. 4(d), we can see that the long MFP phonons (from 500 to 1400 nm) in the regularly bonded bilayer graphene do not contribute too much to the overall thermal transport. However, from Fig. 5(d), we can see that the long MFP phonons (from 500 to 1400 nm) contribute about 30% to the overall thermal conductivity. The above SED results are consistent with the non-monotonic thermal conductivity results shown in Fig. 3.

Furthermore, the comparison of the frequency dependent phonon lifetime and square of phonon group velocity of regularly bonded bilayer graphene between interlayer bonding density of 6.25% and 15% is shown in Fig. 6. We can see that, compared with bonding density of 6.25%, the phonon lifetime of bonding density of 15% is largely reduced. However, the difference of phonon group velocity between the two bonding densities is very small. The previous results have shown that the augmentation of the square of phonon group velocity is the dominant mechanism for the enhanced thermal conductivity of the regularly bonded bilayer graphene compared to the randomly bonded bilayer graphene. From Fig. 6 we can see that, comparing the regularly bonded bilayer graphene between different bonding densities, the change of phonon lifetime, instead of the change of group velocity, is the main reason for the non-monotonic thermal conductivity dependence shown in Fig. 3.

Furthermore, we analyzed the phonon mode localization by calculating the participation ratio *p*_λ_, defined for each mode λ as^30^,^31^,^32^,^33^,^34^


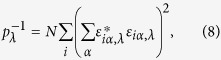


where *i* sums over all the atoms studied, *α* is a Cartesian direction and sums over *x*, *y*, and *z*, *ε*_*iα,λ*_ is the vibrational eigenvector component corresponding to the *λ*^th^ normal mode, and *N* is the total number of atoms. The participation ratio describes the fraction of atoms participating in a particular phonon mode and, hence, it varies between *O*(1) for delocalized states to *O*(1/*N*) for localized states. Figure 7(a) shows the comparison of the phonon participation ratio of the sp^3^ bonded bilayer graphene between random and regular bonding configuration with the same interlayer bonding density of 6.25%. It can be seen that when the phonon frequency is lower than 2.5 THz, the participation ratio for the randomly and regularly bonded bilayer graphene are nearly the same, while for the higher frequency, the regularly bonded bilayer graphene has a significantly larger participation ratio than the randomly bonded structure throughout the whole frequency range, suggesting that the high frequency phonon modes dominate the enhancement of the thermal conductivity of regularly bonded bilayer graphene compared to the randomly bonded bilayer graphene. This is consistent with results of Fig. 4. Further analysis was provided by the mode weight factor^32^,^33^,^34^ defined as


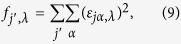


where the prime denotes that sum over *j* (atoms) and is alternatively restricted to the sp^2^ bonded C atoms and the sp^3^ bonded C atoms. Consequently the sum of the mode weight factors in the whole bilayer graphene structure should be equal to unity, i.e.,
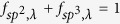
. As 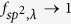 (or 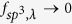), the mode λ tends to become more localized at sp^2^ bonded C atoms and vice versa. Figure 7(b) shows the comparison of the phonon mode weight factor of the sp^3^ bonded bilayer graphene between random and regular bonding configuration with the same interlayer bonding density of 6.25%. Unlike the phonon participation ratio, there is no obvious difference for the mode weight factors of sp^2^ and sp^3^ bonded C atoms, suggesting that the phonon mode weight factor may only depend on the interlayer bonding density, not on the detailed interfacial topological arrangements.

To analyze the mechanisms for the non-monotonic interlayer bonding density dependent thermal conductivity of the regularly bonded bilayer graphene, we further calculated the phonon participation ratio and mode weight factor for the regularly bonded bilayer graphene with different bonding density. The comparison of the phonon participation ratio between bonding density of 5%, 6.25%, 8.75%, 12.5% and 15% is shown in Fig. 8(a), and the inset shows the bonding density dependence of the averaged participation ratio. We can see clearly that the trend of the phonon participation ratio changing with the bonding density is consistent with the thermal conductivity results shown in Fig. 3, i.e., the participation ratio peaks also appear at the bonding density of 6.25% and 12.5%. For the bonding density near these two peaks the participation ratios are largely reduced. Furthermore, the comparison of phonon mode weight factor of the regularly bonded bilayer graphene between interlayer bonding density of 6.25% and 8.75% is shown in Fig. 8(b). It can be seen clearly that with increase of interlayer bonding density, the phonon mode weight factor of 8.75% interlayer bonding density of the sp^3^ bonded C atoms is larger than that of 6.25% interlayer bonding density. The above mode weight factor results combining with that of Fig. 7(b), indicate that the phonon mode weight factor does not depend on the detailed interlayer topological configuration, but only depends on the interlayer bonding density, and the role of the interlayer phonon coupling becomes stronger with the increase of interlayer bonding density.

## Conclusion

To summarize, equilibrium molecular dynamics simulations were performed to investigate the effect of interlayer sp^3^ bonding density on the thermal conductivity of bilayer graphene, especially the results of the randomly and regularly bonded bilayer graphene structures were compared in detail. The thermal conductivity of randomly bonded bilayer graphene decreases monotonically with the increase of interlayer bonding density, which follows the same trend as that obtained in literature. However, for the regularly bonded bilayer graphene structure, we observed the unexpectedly non-monotonic interlayer bonding density dependence of thermal conductivity. The phonon spectral energy density, participation ratio and mode weight factor analysis were performed to explore the underlying mechanism for this counter-intuitive phenomenon. We found that the lifetimes of low-frequency (<5 THz) phonons for randomly and regularly bonded bilayer graphene are nearly the same, which is consistent with the general knowledge that the low-frequency phonons are not sensitive to the detailed interfacial bonding configuration, considering that the characteristic size of the covalent bonding is on the order of only a few angstroms, which is much shorter than the typical wavelength of the low frequency phonons. In contrast, for medium-frequency (5–30 THz) and high-frequency (30–50 THz) phonons the lifetimes in the regularly bonded bilayer graphene are greatly larger than that of randomly bonded structure, especially for the medium-frequency phonon modes which have a rather large contribution to the higher thermal conductivity of the regularly bonded bilayer graphene than the randomly bonded structure. By evaluating the relative importance of the contributions of phonon lifetime and square of phonon group velocity, we conclude that, compared with random bonding, the much higher thermal conductivity of the regularly bonded bilayer graphene is mainly due to the large enhancement in the group velocity of the phonon modes in the medium-frequency range (5–30 THz). In addition, we observe that the thermal conductivity of bilayer graphene depends on not only the interlayer bonding density, but also the detailed topological arrangement of the interlayer sp^3^ bonds. We find that, comparing the regularly bonded bilayer graphene between different bonding densities, the change of phonon lifetime, instead of the change of group velocity, is the main reason for the non-monotonic thermal conductivity dependence. We also characterize the phonon mode localization by calculating the participation ratio and provide evidence that the participation ratio at different bonding density resembles the non-monotonic dependent thermal conductivity. Again this can be correlated with their atomic structure, where the regular bonding density of 6.25% and 12.5% has more homogeneous sp^3^ bonding than that of other bonding densities, which should be responsible for their different phonon band structure. We believe that our analysis provides a comprehensive understanding of the underlying mechanism and the abnormal phenomenon identified in this study can also be extended to other similar bilayer and multilayer structures, that is, the thermal transport properties of bilayer and multilayer structures can be effectively modulated by tuning the detailed arrangement of the interlayer bonding. Other physical properties like electronic and optical properties may also be tuned in the same way and thus such interfacial bonding engineering could offer a robust approach to target desired properties for potential electronic, thermoelectric, photovoltaic, and opto-electronic applications.

## Computational Methods

### Molecular dynamics simulation

**Figure 1:**
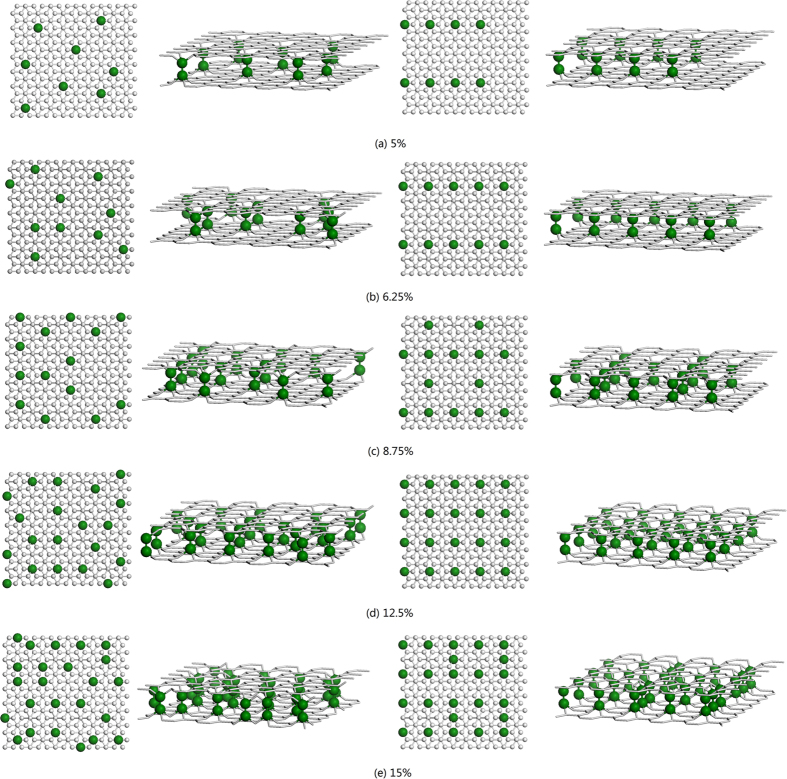
Top and side view of bilayer graphene with interlayer sp^3^ bonding. The left two panels are for random bonding and the right two panels are for regular bonding. From (**a**–**e**) bilayer graphene with interlayer sp^3^ bonding density of 5%, 6.25%, 8.75%, 12.5% and 15%. Color coding: gray, sp^2^ bonded C atoms; green, sp^3^ bonded C atoms.

**Figure 2:**
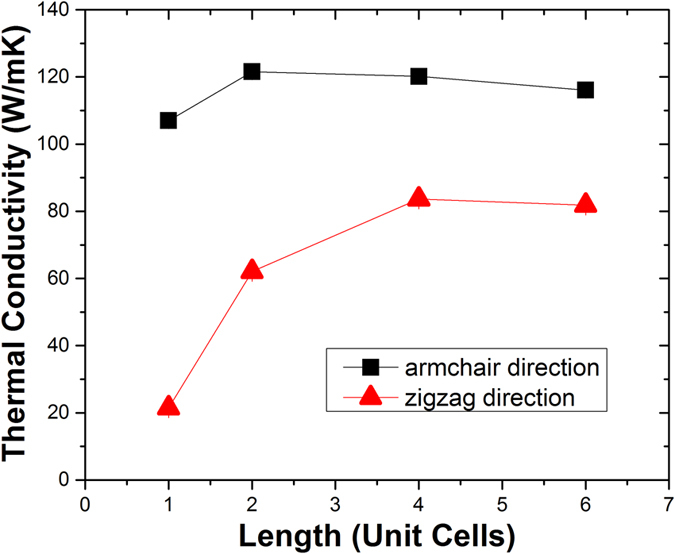
Size effect on the thermal conductivity of regularly bonded bilayer graphene with interlayer sp^3^ bonding density of 6.25%.

**Figure 3:**
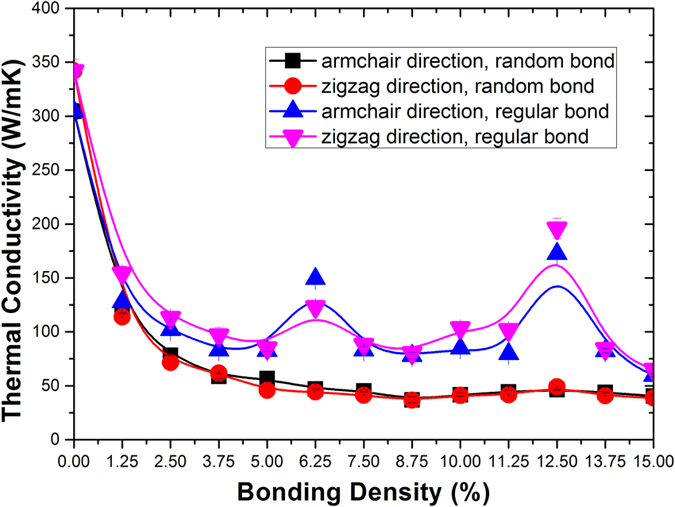
Comparison of the interlayer sp^3^ bonding density effect on the thermal conductivity of bilayer graphene between random and regular bonding configuration. The spline lines are guide for the eyes.

**Figure 4:**
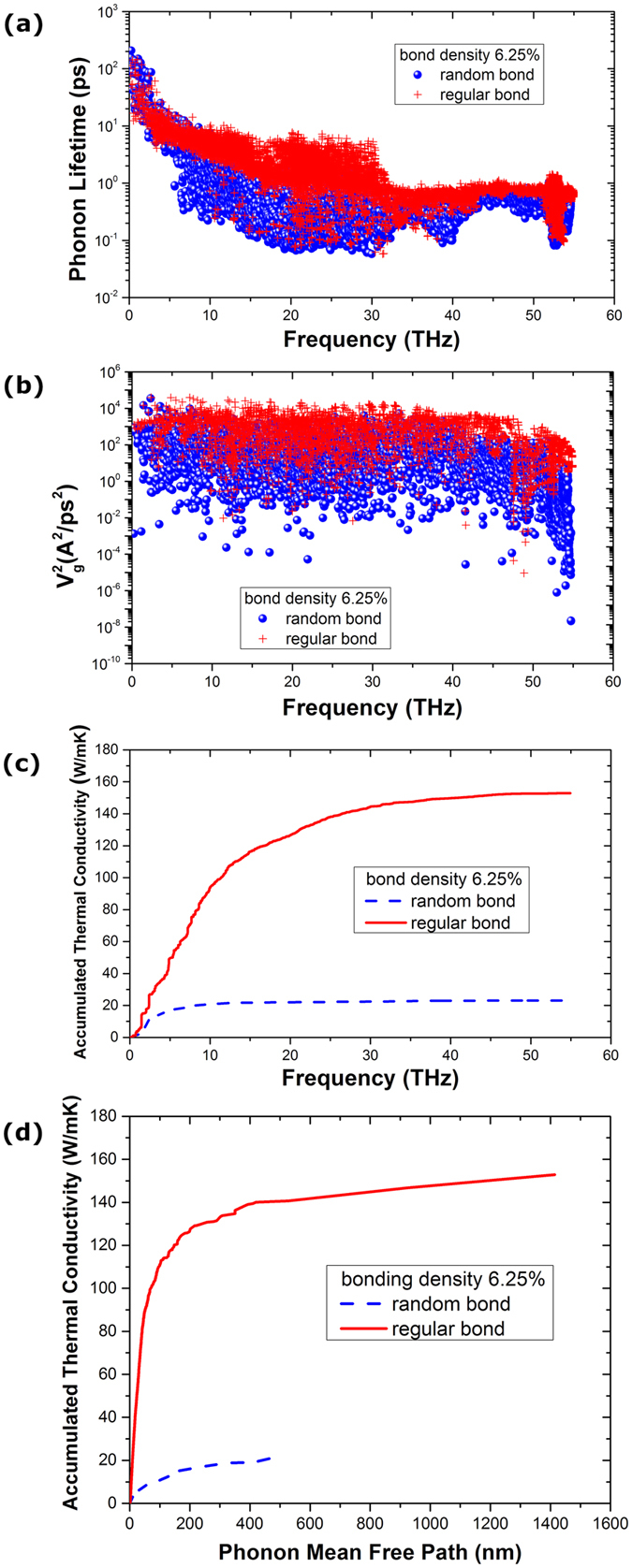
Comparison of the frequency dependent (**a**) phonon lifetime, (**b**) square of phonon group velocity, (**c**) frequency accumulated thermal conductivity and (**d**) mean free path accumulated thermal conductivity of sp^3^ bonded bilayer graphene along armchair direction between random and regular bonding configuration (interlayer bonding density 6.25%).

**Figure 5:**
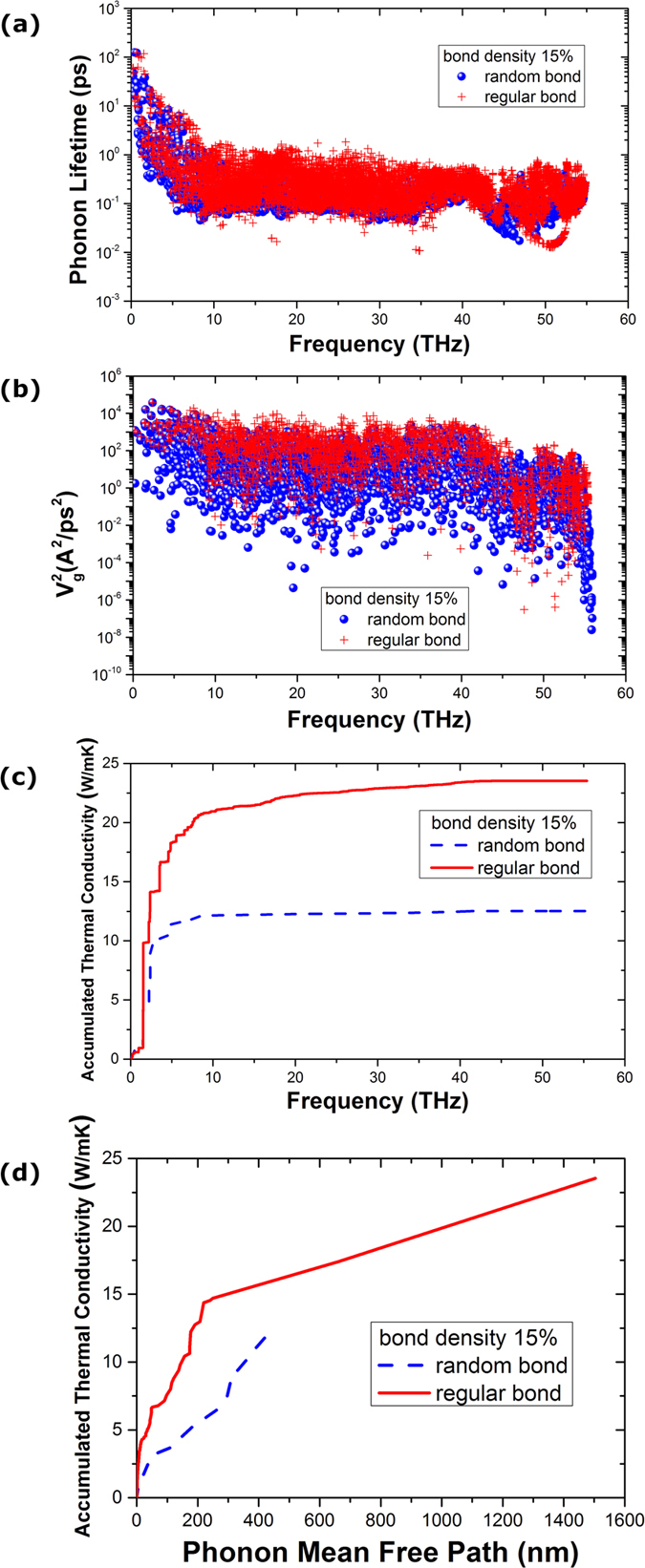
The same as Fig. 4 for the case of interlayer bonding density 15%.

**Figure 6:**
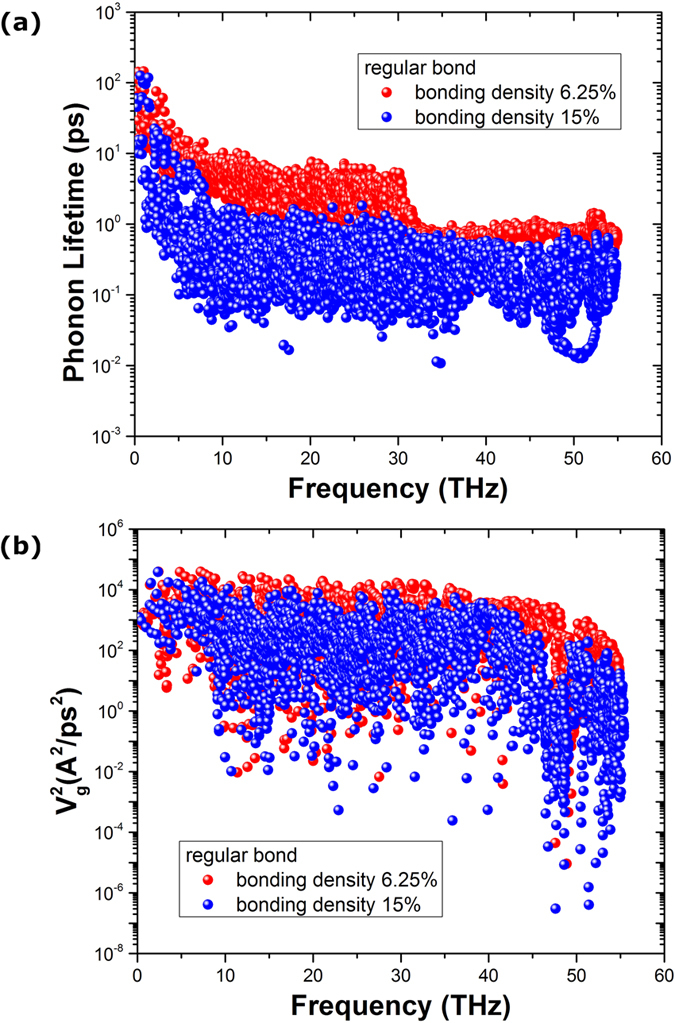
Comparison of the frequency dependent (**a**) phonon lifetime and (**b**) square of phonon group velocity of regularly bonded bilayer graphene along armchair direction between interlayer bonding density of 6.25% and 15%.

**Figure 7:**
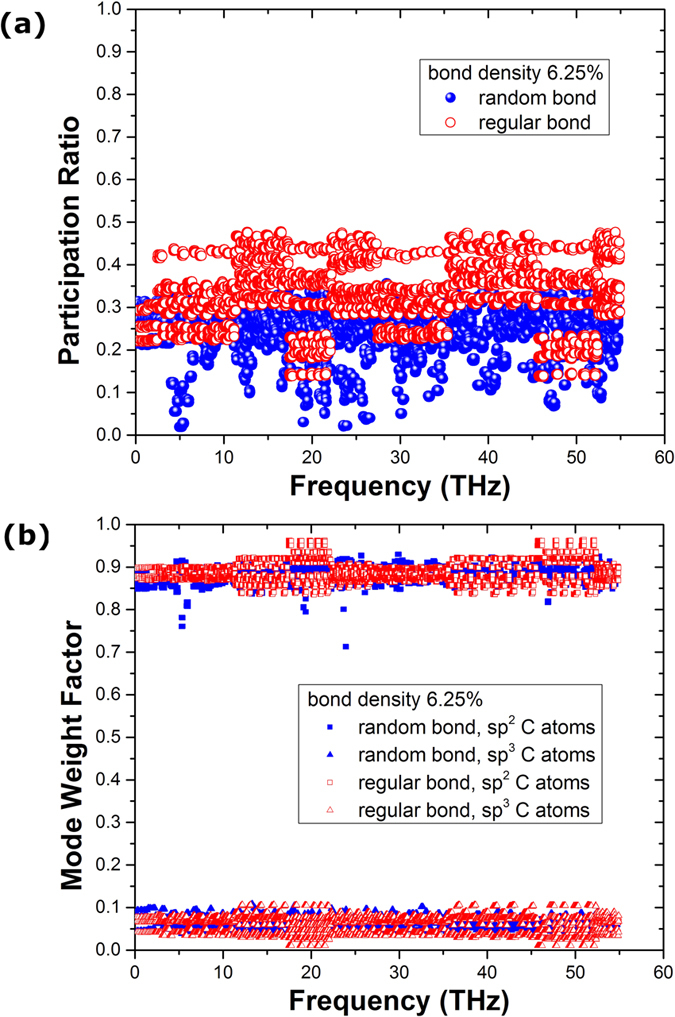
Comparison of the phonon (**a**) participation ratio and (**b**) mode weight factor of the sp^3^ bonded bilayer graphene between random and regular bonding configuration (interlayer bonding density 6.25%).

**Figure 8:**
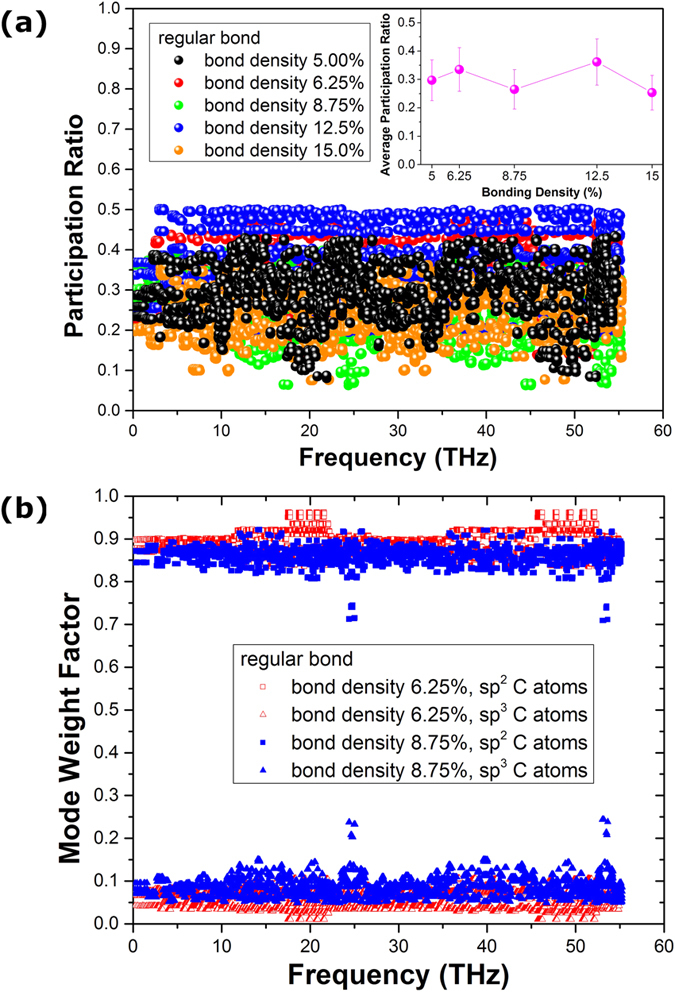
Comparison of the (**a**) participation ratio and (**b**) phonon mode weight factor of the regularly bonded bilayer graphene between different interlayer bonding densities. (Inset) average participation ratio as a function of bonding density.

Our model system of bilayer graphene sheets consist of *n* × *n* supercells with each unit cell constructed by a bilayer honeycomb lattice and composed of 320 carbon atoms, where *n* is ranging from 1 to 6 in our simulations. The structures of a unit cell of bilayer graphene with different interlayer sp^3^ bonding are shown in Fig. 1(a–e) and the initial interlayer distance between the bilayer graphene is set to be 0.34 nm. The interlayer bonding density of 0% corresponds to the bilayer graphene structure without interlayer sp^3^ bonding. To form the sp^3^ bonded bilayer graphene structures shown in Fig. 1(a–e), the inter-layer bonding process is realized as follows: Firstly, the bonded atoms are chosen and moved closer to each other along the out-of-plane direction until the distance between the two bonded atoms reaches 0.142 nm. Secondly, the period boundary condition is applied in the in-plane directions, then the bonded atoms are fixed and all other atoms in the system are relaxed with *NPT* (constant pressure and temperature) ensemble for 100000 steps. Thirdly, the fix on the bonded atoms is removed and the entire system is relaxed with *NPT* ensemble for additional 1000000 steps. Finally, the stable bilayer graphene with inter-layer bonding is achieved. For each model system we have carefully checked the topography and bonding density of the interlayer bonding before and after structure relaxation, and also before and after the later on EMD simulation of calculating thermal conductivity. For all cases we confirm that there is no change for the topological configurations of the interlayer bonds and the interlayer bonding density due to temperature fluctuations. To investigate the effect of the topological configuration of the interlayer bonding on the thermal transport in bilayer graphene, here we constructed both the randomly and regularly distributed bonding structures. In our simulations, we only considered the interlayer bonding density lower than 15% but the thermal conductivity of nanostructures is very sensitive to the component of the structures^35^. So, if possible, higher interlayer bonding density should also be investigated. In fact, we have tried the bonding density higher than 15% and found that a lot of additional bonding will appear. The reason for this phenomenon is that, for high bond density model too many bonded atoms are distributed in a small region and this will force the atoms that are originally un-bonded to come closer and finally bond with each other. Once they are bonded, the bond will not break at the temperature we considered. These additional bonding will definitely affect the accuracy of our results. We therefore did not continue the simulation for higher bonding density.

The adaptive intermolecular reactive empirical bond order (AIREBO) potential^36^ was used to describe the C-C interatomic interactions in the model system. This potential has been widely used to investigate the thermal transport properties of carbon materials, e.g., single polyethylene chains^37^, carbon nanotubes (CNTs)^38^, graphyne sheets^39^ and nanotubes^23^, and graphene^40^ systems. Note that in our simulations, the Lennard-Jones interaction term of the AIREBO potential was turned on and the cutoff distance was set to be 1.02 nm, and the torsion term of a four-body interaction was turned off. Based on our previous simulation results^41^, the torsion term has a relatively small contribution to the overall thermal transport properties of the model system compared to the two-body and three-body interactions. Another reason for turning off the torsion term is that it can largely save simulation time. Actually, the same treatment has been already used in many previous studies, e.g. refs 42, 43. All MD calculations were performed using the large-scale atomic/molecular massively parallel simulator (LAMMPS)^44^ with a small enough timestep of 0.1 fs throughout to ensure the energy conservation. Periodic boundary conditions are used in *x* and *y* directions (in-plane), and a free boundary condition is used in *z* direction (out-of-plane). In the first stage of the MD simulations, we relaxed the system at constant atmospheric pressure and room temperature (300 K) for 0.1 ns, using the Nosé-Hoover thermostat and barostat^45^,^46^. After *NPT* relaxation, we continued to relax the system with *NVT* (constant volume and temperature) ensemble for 0.1 ns using the Nosé−Hoover thermostat and *NVE* (constant volume without thermostat) ensemble for additional 0.1 ns.

### Thermal conductivity calculation

Following equilibration, the thermal conductivity of bilayer graphene was estimated by the Green-Kubo method^47^


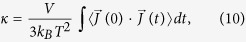


where *V* is volume, *k*_*B*_ is the Boltzmann constant, *T* is temperature, 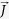 is heat flux, 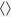 denotes the time average. The heat flux 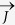 was calculated by


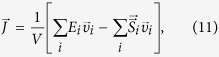


where *E*_*i*_ is the total energy (kinetic and potential) of atom *i*, 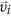 is the velocity of atom *i*, 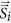 is the symmetric stress tensor of atom *i* defined as^44^


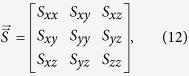


with


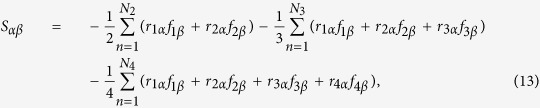


where *α* and *β* denote the *x*, *y* or *z* direction. The three terms in the definition of *S*_*αβ*_ define the contribution from two-body, three-body and four-body interaction, respectively. The thickness of bilayer graphene was chosen as 0.68 nm in calculating the system volume, which is equal to two times of the van der Waals diameter of the C atom^48^. To achieve a good convergence for the thermal conductivity of bilayer graphene, we took every successive 50 ps (a typical autocorrelation time) of heat current data as different samples and calculated the thermal conductivities of these samples. Furthermore, using the above method we run 5 independent simulations with different initial atomic velocities. Typically, for each simulation we used 5–7 ns of heat current data to calculate the thermal conductivity. We first calculated the average thermal conductivity of all the heat current samples in each simulation, and then the final thermal conductivity result was taken as the average over the 5 runs for correlation time between 20 ps and 50 ps.

### Phonon spectral energy density analysis

We used a unit cell of bilayer graphene structure with 2.13 nm × 1.97 nm (320 atoms) as the input for the phonon normal mode calculation. And limited by the large unit cell, we only used a 4 × 4 supercell (5120 atoms) for the phonon spectral energy density analysis. To achieve enough frequency range for bilayer graphene, we sampled the atomic velocities with a time interval of 9 fs, and the max phonon frequency we can achieve is about 55.56 THz which is a little larger than the max frequency of bilayer graphene. It is worth pointing out that, strictly speaking, there is no periodicity for the randomly distributed bonding structure and the wavevector-related phonon information cannot be obtained for the phonon spectral energy density calculations, here we just distributed the atoms irregularly in the unit cell to achieve the randomly bonded bilayer graphene structure.

## Additional Information

**How to cite this article**: Zhang, X. *et al*. Robustly Engineering Thermal Conductivity of Bilayer Graphene by Interlayer Bonding. *Sci. Rep.* **6**, 22011; doi: 10.1038/srep22011 (2016).
